# When information does not suffice: young people living with HIV and communication about ART adherence in the clinic

**DOI:** 10.1080/17450128.2015.1128581

**Published:** 2015-12-30

**Authors:** S. Bernays, S. Paparini, D. Gibb, J. Seeley

**Affiliations:** ^a^ Department of Social and Environmental Health Research, London School of Hygiene and Tropical Medicine, London, UK; ^b^ MRC Clinical Trials Unit at University of College London, London, UK; ^c^ Department of Global Health and Development, London School of Hygiene and Tropical Medicine, London, UK & MRC/UVRI Uganda Research Unit on AIDS, Entebbe, Uganda

**Keywords:** Adolescents, adherence, disclosure, qualitative, communication, clinical care

## Abstract

Despite mounting evidence recommending disclosure of human immunodeficiency virus (HIV) status to young people with perinatally acquired HIV as a central motivating factor for adherence to antiretroviral therapy, many young people continue to experience disclosure as a partial event, rather than a process. Drawing from two longitudinal, interview-based qualitative studies with young people living with HIV (aged 10–24) in five different countries in low and high income settings, we present data regarding disclosure and information about HIV in the clinic. The article highlights the limits of discussions framing disclosure and patient literacy, and young people’s reluctance to voice their adherence difficulties in the context of their relationships with clinical care teams. We suggest that a clinician-initiated, explicit acknowledgment of the social and practical hurdles of daily adherence for young people would aid a more transparent conversation and encourage young people to disclose missed doses and other problems they may be facing with their treatment. This may help to reduce health harms and poor adherence in the longer-term.

## Introduction

Rates of adherence to antiretroviral therapy (ART) amongst young people living with human immunodeficiency virus (HIV), particularly adolescents (aged 10–19), are lower than in adult counterparts (Lowenthal et al., 2014). This represents a significant public health challenge given the size of the global paediatric HIV population (over 3 million, of which over 2 million are adolescents; Joint United Nations Programme on HIV/AIDS (UNAIDS), 2014); low treatment access for adolescents (around 24%; UNAIDS, 2014); the extra years of treatment-taking faced by young people with perinatally acquired HIV and restricted access to second and third line treatment options in resource-limited settings (Lowenthal et al., 2014; Vreeman, Gramelspacher, Gisore, Scanlon, & Nyandiko, 2013).

A complex interplay of social and developmental factors affect ART adherence for this population. These include, but are not limited to, the social risks associated with treatment-taking and deductive disclosure of their HIV status, and young people’s limited agency in terms of their behaviours and living environments (Bernays, Jarrett, Kranzer, & Ferrand, 2014; Bernays, Seeley, Rhodes, & Mupambireyi, 2015; Denison et al., 2015; Kawuma, Bernays, Siu, Rhodes, & Seeley, 2014; Lowenthal et al., 2014).

Another central factor shaping adherence behaviours in young people is knowledge of their own HIV positive status. Quantitative and qualitative research in all income settings shows the positive effects that telling young people their status has on ART adherence, mental health and onward disclosure by young people to their sexual partners and others (Cluver et al., 2015; Evangeli & Foster, 2014; Fetzer et al., 2011; Vreeman et al., 2010, 2013). Disclosure tends to be less frequent and happen later in resource-limited settings (Vreeman et al., 2013), yet rates of adherence amongst young people are higher in some of these settings than in comparable populations in high-income countries (Kim, Gerver, Fidler, & Ward, 2014). Contradiction in these findings would suggest that, although conceived as a critical step to improve or sustain adherence, disclosure should not be interpreted as *sufficient* to ensure adequate adherence.

Despite the emphasis in global and national clinical guidelines (WHO, 2011), disclosure continues to be postponed beyond the recommended age (Vreeman et al., 2013). Research has emphasised carers’ anxieties surrounding disclosure to their children, (Kyaddondo, Wanyenze, Kinsman, & Hardon, 2013; Mandalazi, Bandawe, & Umar, 2014; Vreeman et al., 2015), but talk about HIV between young people and their clinicians is also limited to a minimum monitoring of ART and physical symptoms (Bernays et al., 2015). Young people may thus experience disclosure as an incomplete event, rather than as part of a fuller discussion over time as appropriate (Vreeman et al., 2013). Ongoing, more effective communication with young people to help sustain ART adherence and condition management is crucial to protect their health as they grow up. It would also improve the challenging public health implications of poor adherence, including the prevention of onward transmission (Kim et al., 2014).

In this brief article, we present data from two large, longitudinal qualitative studies with young people living with HIV in five different countries. We address their experiences of adherence post-disclosure and, especially, their motivations for missing treatment and avoiding seeking help in the clinic for adherence issues.

## Methods

We draw on two multi-country longitudinal, mixed qualitative methods studies with children and young people growing up with HIV. Both studies were conducted in HIV clinics within international clinical trials. Though different in foci and objectives, both studies collected data on young people’s experiences and challenges in relation to disclosure, adherence, and reporting missed doses. To be included in both studies, participants had to have been aware of their HIV status for at least 6 months prior to the start of recruitment into the qualitative study.

One qualitative study is a sub-study of a randomised control trial (BREATHER; 2011-ongoing) exploring the efficacy of short cycle therapy (SCT) on Efavirenz-based regimens (5 days on, 2 days off treatment). The focus of the qualitative study is the acceptability of the intervention and the contextual challenges and facilitators to maintaining adherence. All young people recruited into the BREATHER trial in the UK, Ireland, Uganda and the USA aged 10–24 years were eligible to participate, subject to the appropriate consents and self-awareness of HIV infection. Overall 102 individual in-depth interviews have been conducted (to date) with 43 young people (Table 1): 26 young people were recruited in Uganda from one clinic (Joint Clinical Research Centre, Kampala); seven in the UK and Ireland from three clinics (hospitals in London, Nottingham and Dublin); and 10 in the US from one clinic (St. Jude’s Children’s Research Hospital, Memphis). The qualitative sample in each site reflects the diversity of the trial population (Table 1). To our knowledge, participants in the US sample all acquired HIV horizontally, whilst the remaining participants from UK, Ireland and Uganda were all perinatally infected with HIV. We present here data from the repeat in-depth interviews with young people over the course of the trial (three phases of data collection in Uganda, two in the UK, Ireland and USA). Due to the small sample sizes in the UK and Ireland, reports are combined to avoid identification of participants.

**Table 1.** Qualitative samples’ overview.

Country	*n*	Male	Female	Age range	Age median	
**BREATHER TRIAL**						
Uganda	26	12	14	11–22	17	
UK (& Ireland)	7	5	2	12–17	16	
USA	10	9	1	18–22	21.5	
**Total**	**43**	**26**	**17**	**11–22**	**17**	
				**Aged** **11 (*n*)**	**Aged 12 (*n*)**	**Aged 13 (*n*)**
**ARROW TRIAL**						
JCRC, Kampala, Uganda	26	9	17	7	9	10
PIDC/ Baylor, Kampala, Uganda	26	11	15	12	6	8
MRC/ UVRI, Entebbe, Uganda	26	13	13	9	8	9
University of Zimbabwe, Harare, Zimbabwe	26	12	14	8	9	9
**Total**	**104**	**45**	**59**	**36**	**32**	**36**

Interviews in the earlier phases reflected the beginning of the trial, participants’ views on taking part, their first thoughts on the intervention, and an initial discussion about their experiences of growing up with HIV and on ART, including broader social and family circumstances. In the subsequent phases, the interviews addressed ongoing questions of trial participation, therapy changes (for those randomised to SCT), experiences of clinical care and any developments in the issues discussed in the early phase of the study. The longitudinal design allowed researchers to build rapport with young people who felt able to share in more detail their reflections on their condition, treatment and participation in both the trial and the qualitative research study.

The second qualitative study (2011–2013) was embedded within the ARROW randomised controlled clinical trial (assessing two different management strategies for monitoring first line anti-retroviral treatment for paediatric HIV). The trial research questions were not a substantive focus of the qualitative study, which explored more broadly how experiences of growing up with HIV and ART interplayed with everyday life. Repeat in-depth interviews were carried out with 104 children living with HIV (aged 11–13 years) (Table 1). Fully detailed methods of the ARROW study have been previously reported elsewhere (Bernays et al., 2015).

Both studies were granted the necessary national ethical approvals. In both studies audio-recorded data were transcribed verbatim and where appropriate translated into English. The studies adopted a grounded analytic approach to thematic analysis, using systematic case comparison and negative case analysis throughout (Strauss & Corbin, 1990). First-level coding drew upon a combination of a priori themes reflected in the study topic guide and inductive or in vivo codes (Charmaz, 2006), whilst second-level coding sought to break down first-level coded data into smaller units, which also involved moving from codes which operate at the level of participant description and meaning to concept-driven categories. This process is similar to moving from ‘open’ to ‘axial’ to ‘selective’ coding in grounded theory (Strauss & Corbin, 1990). Core themes across both studies included: incomplete disclosure; silence surrounding HIV; adherence as a form of disciplining; and reputation management.

## Findings

### Disclosure of their HIV status

Regardless of age, disclosure was experienced by many of the young people in our studies as a one-off event, frequently framed in stark medical terms: participants were informed that HIV is a virus with the potential to lead to terminal illness if not treated, and this was why they had been taking their drugs (if they had been taking ART prior to disclosure) and should continue to do so. Little further information was shared and questions were commonly foreclosed. Disclosure was rarely followed up by any other conversation about HIV, so participants wondered about important issues such as the circumstances of their own (and their parents’) infection, how to manage information about their status in the household, school and wider community, or the implications of HIV for their own future relationships and plans.

Some young people responded by doing research of their own, on the Internet for example, and only a few asked questions to their carers, other young people, other adults, clinical care staff or at times the qualitative researchers themselves. The majority reported not feeling comfortable asking questions, hence relied on piecemeal information from which they tried to make sense of their own situations. Amidst this characteristic silence, additionally described in a related article (Bernays et al., 2015), it was nonetheless made clear to them that they should adhere to their daily medication at all times, lest they fall ill or die.

Thus, all the young people knew they were living with HIV but not many knew what that meant, beyond being acutely aware that it involved taking pills every day for the rest of their life.

*No I haven’t spoken to my psychiatrist since* [finding out HIV status] *(…) She talked to me and then she said, any questions about that? No*, [I] *just left it at that. **Did you talk about it with your mum?** No. **So you’ve never spoken about it with your mum?** No*. (Lenny, 12, UK & I, BREATHER)

### Patient literacy

This incomplete experience of disclosure nonetheless did, in many cases, produce a strong, moralised commitment to ART. Although treatment ‘literacy’ levels varied, for the most part due to age, participants’ understanding that they were meant to take their treatment as prescribed was evident. Yet an emphasis on the absolute need to never miss a dose did not engage with ‘how’ they were meant to always be able to take their treatment. Many participants expressed frustration that healthcare staff and others did not appear to appreciate ‘how difficult it is’ (Mark, 11, ARROW, Uganda).

Significant awareness of the risks involved in *not* taking their medicines as well as a capacity to *adapt* their treatment-taking to their day-to-day activities, revealed participants’ appreciation that they were ‘deviating’ from the clinician’s instructions.

Knowledge of what was expected of them in terms of adherence did not necessarily equip them to deal with problems when trying to adhere. Instead, such awareness at times had the reverse effect of creating anxiety when they were not able to take their treatment for reasons within or beyond their control.

*Because I’m at* [work place] *I’ve been missing it more than I would usually miss it. I’d probably say five or six times and then it’s, I hate that so much. **And what’s more usual?** More usual would be at least, I would say out of every three weeks it would probably be I miss like twice or three times. But since I’ve been at* [work place] *I’ve been missing a lot lately*. (Jason, 20, US, BREATHER)

Participants very frequently elected not to tell anybody about missed doses or any adjustments to their regimens, such as times of day, they had made on their own. In absence of questioning, symptoms or obvious changes in their clinical markers, the fear of admitting their adherence-related problems could often override the need to talk to clinicians. The outcome of this calculation was, for the majority, silence about their difficulties and a pronounced sense of the need to manage their treatment challenges alone.

Young people had little reason to expect that their ‘failure’ to adhere would be understood, since nobody had made reference to the fact that they may have good reason to find it hard to take treatment as prescribed. Any support, sanctioning or discussion about their adherence struggles was thus done reactively, for example when they were ‘caught’ skipping doses or changes showed in their clinical monitoring. This almost inevitably failed to create the conditions to ameliorate some of the issues they were facing, as conversations started from the perspective of a ‘problem’ that had already arisen, rather than from an explicit acknowledgement that problems *might* arise. Hence there was little proactive communication about the kinds of strategies that could be put in place to avoid harm being caused to their health by non-adherence.

Instead, young people talked about how clinicians reiterated to them the necessity to continue to adhere as prescribed, with what appeared as little or no acknowledgement of the broader conditions that had contributed to missed doses and interruptions in the first place.

***Did you come back to the clinic after missing a dose (…)?*** *Yes. **What happened when you came back to the clinic? (…)** I brought back the pills and explained to them. **Which healthcare worker did you tell?** They asked me that, ‘Why do you have drug balances?’ And, they said that since you have explained to us, there’s no problem. But I shouldn’t do it again*. (Songs, 12, Uganda, ARROW)

At repeated points in time, then, opportunities emerged to open up the clinical conversation about young people’s pragmatic, social and personal struggles with ART. Yet these were very rarely taken up until the next problematic event. The burden of responsibility continued to be placed on young people to ensure their adherence regardless of obstacles and concerns, hence they learnt to keep quiet about non-adherence unless, and until, talk was unavoidable.

### Managing reactions: from scolding to praising

Young people in both our studies reported being scolded by health care staff and carers when they did not take their pills. The content and harshness varied, but the overall tone of reproach for what was framed as their individual failure to adhere, to act as prescribed, was a common feature of the narratives of our participants.

In Zimbabwe and Uganda, pressure was also put on young people to maximise benefits of available ART. This was done by emphasising their need to be grateful for the care they receive, but also to be mindful of the many peers who lack access to the same life-saving drugs.

*There are some healthcare workers who can say that if you don’t want to take medicine and you waste it, others want to take it. You waste drugs, and they say that as they quarrel. And there, your mother will be angry with you*. (Anita, 12, Uganda, ARROW)

As well as the ‘shame’ of not taking their pills, young people were also reminded about the risks of missing doses through reference, in the cases where they had been orphaned, to the death of their biological parents. Memories of childhood illness were further used to urge young people to take their pills, as were examples of the fate of other people living with HIV who were suffering from ill health (e.g. on TV, in the papers, in the clinic or in the community). However, these displays of the perils of non-adherence were rarely contextualised, for example in relation to the availability of medication. Non-adherence was thus inevitably presented to them as the end-product of individual *choice* rather than a complex interplay of circumstances.

The younger participants were particularly concerned about being reprimanded by the healthcare workers, yet the perception that admissions of non-adherence would incur a negative reaction was common in both studies across age-ranges. Young people *expected* that non-adherence would be sanctioned, rather than understood, which made it increasingly difficult to voice any problems, for fear of the repercussions in the clinic and the knock-on effect at home. For example, when asked why a child might not tell a healthcare worker about missing or forgetting ARVs, John said: ‘He knows that they are going to scold him’ (13, Zimbabwe, ARROW), whilst Job said: ‘because at the time that he will be scared, he will be thinking that he will be beaten’. (13, Uganda, ARROW)

At the opposite end of the spectrum from scolding, praising young people for being ‘exemplary adherers’ could also have a problematic impact on their ability to disclose problems with treatment or instances of non-adherence: ‘I’m scared of disappointing people’ (Mike, 20, USA, BREATHER). This is because young people felt that, by failing to live up to their own reputation for having optimum adherence, they would damage their relationships with clinicians, for whom they felt gratitude.

*I’m still a bit scared and like maybe I shouldn’t have missed that dose. **Would you tell anyone?** No, I don’t think I’d tell anybody, no **(…)** Yeah, it is a bit tough. I suppose ‘specially to tell family and maybe the consultants themselves as well which you think they care about you so much and if you tell them that you’ve missed your dose they might think that you’re giving up on yourself and I don’t know anymore*. (Rob, 15, UK & I, BREATHER)

Scolding and praising are respectively linked to the ideas of young patients as being either ‘failures’ or ‘successes’, with very little space in the middle and no apparent accommodation of shifts and changes in young people’s capacity to take their treatment. Both labels can have a fixing effect that at times produced further silencing of any challenges with ART.

## Discussion

A lack of clinician-initiated acknowledgement of the potential for adherence to be disrupted at times can cause young people to withhold information about missed doses with their healthcare teams. Many of the skipped doses participants talked about in the qualitative studies did not necessarily cause significant harm to their health, or remained undetected by clinical monitoring. However, minor adherence interruptions are being missed and issues may only be detected once they have become more serious and harder to address.

Thus, our studies strongly suggest that information about HIV status or literacy is not sufficient to motivate good adherence over time. Simultaneously, the very knowledge about what is expected of them, reinforced also through scolding or praising, can be a disincentive for young people to disclose non-adherence.

Adherence must be supported by helping young people to develop an appreciation of the wider *implications* of being HIV positive and of taking life-long ART. This includes an explicit and repeated recognition of the fluidity of adherence as it can be affected by factors beyond young people’s control, their changing circumstances, periods of transition, schooling, changes in household composition, or shifts in adherence management (between different carers or from carers to young people).

Rather than setting a fixed standard of adherence with little accommodation for these realities, clinicians could support young people by proactively validating the boundaries of the expectation placed on patients to be responsible to take treatment every day, as prescribed, at all times. A margin for error that is understood by their clinicians may not necessarily be a disincentive for young people to adhere, but could rather incentivise *disclosure of non-adherence* and support-seeking.

Young people might be more likely to express their issues with ART, enabling transparency about the circumstances of their treatment-taking. Solutions could be tailored to optimise adherence in specific instances as they present themselves, rather than a reliance on admonishing young patients not to repeat their ‘mistakes’. A more open discussion about the social environments and relationships that support or hinder young people’s adherence is required, along with a shift away from the problematisation of young people’s behaviour as ‘irresponsible’ in the context of poor adherence.

Although based on a large sample, longitudinal design and multi-country research, our analysis is nonetheless centred on the situation of young people who are receiving care in what are widely recognised as centres of clinical excellence, both in high and low income countries. In doing so, we focus here on what could be conceived of as a selective ‘tip of the iceberg’ sample of young people accessing well-resourced HIV clinics in order to draw attention to how, even within this group, problems with adherence and with discussing non-adherence prevail nonetheless. Although we have suggested ways to improve current clinical communication regarding HIV and ART that would benefit this group, the aim is to provide evidence that might be of help also for those caring for positive young people in more challenging clinical and community settings.
